# Supplementary material for: Saccharomyces boulardii (CNCM I-745) improves intestinal damage in sepsis by remodeling intestinal flora structure
Source: Front Cell Infect Microbiol. 2025 Jul 31;15:1602792. doi: 10.3389/fcimb.2025.1602792 (PMC12350329; doi:10.3389/fcimb.2025.1602792)
Supplement: Supplementary file 1 [file DataSheet1.zip › summary/summary/7_Differential_analysis/4_Manhattan_plot/Group.DW4_vs_DW0/Group.DW4_vs_DW0_manhattan.pdf]

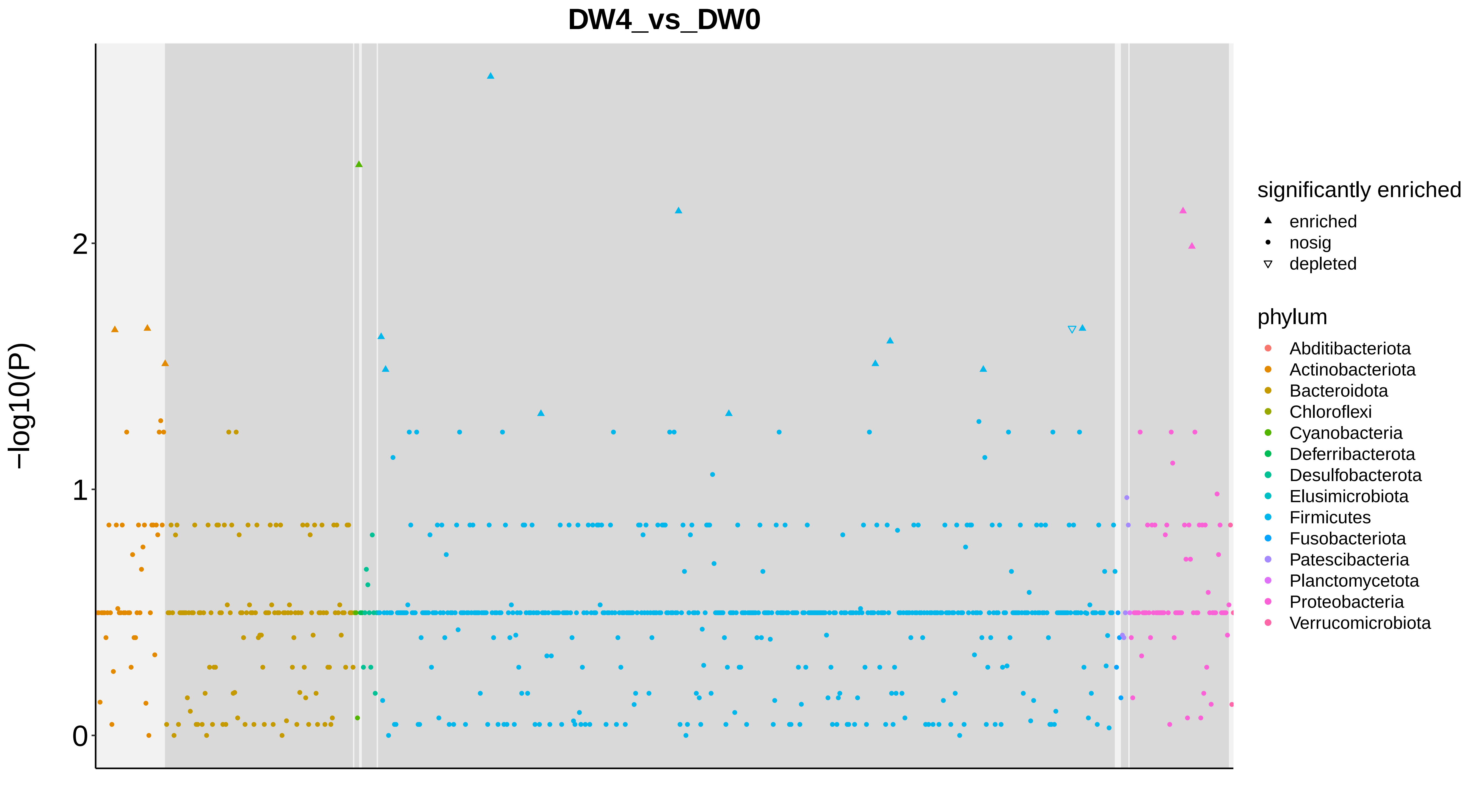
DW4_vs_DW0
−log10(P)
0
1
2
significantly enriched
enriched
nosig
depleted
phylum
Abditibacteriota
Actinobacteriota
Bacteroidota
Chloroflexi
Cyanobacteria
Deferribacterota
Desulfobacterota
Elusimicrobiota
Firmicutes
Fusobacteriota
Patescibacteria
Planctomycetota
Proteobacteria
Verrucomicrobiota
